# Polysiloxane Networks Modified by Nitrogen-Containing Organic Compounds

**DOI:** 10.3390/ijms262211133

**Published:** 2025-11-18

**Authors:** Aleksandra Chechelska-Noworyta, Maria Owińska, Magdalena Hasik

**Affiliations:** Faculty of Materials Science and Ceramics, AGH University of Krakow, al. Mickiewicza 30, 30-059 Krakow, Poland

**Keywords:** polysiloxane networks, cross-linking of polyhydromethylsiloxane, hydrosilylation of amines, functionalized polysiloxanes

## Abstract

Amine-functionalized polysiloxanes, due to the presence of amino moieties, can be used for the extraction of toxic metal ions from wastewater, as supports for metallic catalysts, stabilizers for metal nanoparticles, macromolecular biocides, or as self-healing materials. In the present work, we studied poly(hydromethylsiloxane) (PHMS) networks functionalized with three amines: *N*-allyaniline (Naa), *N*-allylcyclohexylamine (Nach), and *N*-allylpiperidine (Nap). They were prepared using two procedures. The first one was a two-step process in which the previously cross-linked PHMS was reacted with the amine. The second, one-step method involved simultaneous PHMS cross-linking and reaction with the amine. FTIR and ^29^Si MAS-NMR spectroscopic investigations, as well as elemental analysis, allowed us to conclude that the one-step method was more advantageous. It ensured higher PHMS networks functionalization degrees and hindered hydrolysis/condensation of Si-H/SiOH groups side processes, which were related to the basicity of the studied amines and significant in the two-step procedure.

## 1. Introduction

Polysiloxanes are synthetic polymers with a structure based on an inorganic backbone formed by Si-O bonds. They exhibit a number of unique properties, such as high thermal and chemical stability, hydrophobicity, elasticity, high permeability to gases, and physiological inertness [1,2,3,4,5]. Among these polymers, polyhydromethylsiloxane (PHMS) is of particular significance. This linear polysiloxane, as well as its copolymers (e.g., PHMS-DMS, which contains dimethylsiloxane units), have reactive Si-H side groups that are susceptible to chemical modification [6]. This makes it possible to alter the chemical structure of these polymers by incorporating a variety of organic functional groups. Furthermore, the presence of reactive Si-H moieties enables cross-linking of polymer chains, transforming a liquid, soluble polymer into solid, insoluble materials [7]. These facts exert a significant influence on physical and chemical properties and thus on the applications of this group of materials used as coatings, sealants, greases, pharmaceuticals, and medical materials [5,8,9]. Thermal transformations of organosilicon networks lead to SiOC ceramic materials [10].

Hydrosilylation reaction is an efficient and widely used method for the introduction of functional groups into polysiloxanes, as well as for cross-linking of polysiloxane chains [1]. This reaction involves the addition of Si-H groups to multiple carbon–carbon or carbon–heteroatom bonds in the presence of a catalyst [11]. In the hydrosilylation of carbon–carbon double bonds, a process most suitable for the preparation of organofunctional siloxanes (due to the formation of Si-C bonds) [6,12], two Pt catalysts: Speier’s (hexachloroplatinic acid) and Karstedt’s (a complex of Pt^0^ and 1,1,3,3-tetramethyl-1,3-divinyldisiloxane), are very efficient and mainly used [7,13]. In the literature, there are many reports on the successful incorporation of a variety of organic moieties into polysiloxanes by hydrosilylation. Among the most important organic groups we can distinguish: epoxy and other oxygen-containing groups (e.g., acrylic and methacrylic ester or acid and polyether moieties), fluoroalkyl groups, as well as amine and other nitrogen-containing groups (e.g., carbazolyl, phthalocyanine or cyclam side groups) [12]. Hydrosilylation reaction is also an important method for the preparation of three-dimensional polysiloxane networks. The formation of cross-linked systems occurs as a consequence of the addition reaction and creation of covalent bonds between reactive groups present in the polymer (e.g., Si-H moieties in PHMS) and suitable reactive groups (e.g., -CH=CH_2_ groups) present in the low-molecular cross-linking agent. As cross-linking agents, linear, cyclic, or branched multifunctional siloxanes [14,15,16], polyhedral oligosilsesquioxanes (POSS) [17], as well as organic compounds, e.g., dienes [18], can be used. The structures and properties of final networks obtained by hydrosilylation reactions depend on the type of reagents used and the molar ratio of reactive functional groups. It is therefore possible to obtain polysiloxane networks with different cross-linking densities, including systems with low cross-linking density using a greater excess of Si-H groups from the polymer [14]. This is significant because Si-H groups allow for the post-modification of such materials [19].

Amine-functionalized polysiloxanes exhibit enhanced chemical and physical properties resulting from the combination of the unique properties of both siloxane backbone and organic moieties. Therefore, they can act as ligands for metal ions or particles and can be used for the extraction of toxic metal ions from wastewater [20], as supports for metallic catalysts [21,22], or as stabilizers for metal nanoparticles [23]. Amino- and ammonium moiety-containing polysiloxanes are an important class of macromolecular biocides [24,25,26,27,28]. In recent years, self-healing materials based on amine-functionalized polysiloxanes have gained increasing attention [29].

Polysiloxanes modified by amino groups can be prepared by polymerization of a functionalized monomer or by post-functionalization of the previously synthesized polymer containing appropriate reactive groups. The incorporation of amine moieties into siloxanes can be achieved through various methods. One of the possible approaches involves hydrolytic condensation or polycondensation of alkoxysilanes containing incorporated nitrogen moieties [20,21,22,24,30,31]. *N*-methylaza-2,2,4-trimethylsila-cyclopentane can serve as the source of aminoalkyl units in siloxanes [23]. Other methods rely on the transformation of functional groups (e.g., azide [32,33]) previously introduced into siloxane molecules. Additionally, if suitable monomers are available, ring-opening polymerization of cyclic siloxanes containing nitrogen atoms can be performed to obtain modified polymers [24].

Numerous published reports concern linear polysiloxanes bearing reactive units that allow their post-functionalization. Reactions involving epoxide ring opening in the presence of amines [34,35,36,37], substitution reactions on functional groups such as 3-chloropropyl [24,25,26] or 3-mercaptopropyl [26], as well as thiolene “click reaction” [28] allow for the incorporation of amino groups into the polymer. Another strategy for introducing amino functionality into polysiloxanes is hydrosilylation. It should be noted that reactions of unsaturated amines in the presence of Pt catalysts are challenging, as they may be accompanied by undesired side processes that result in polymer cross-linking [13] or catalyst poisoning [7]. However, there are reports in the literature on successful direct hydrosilylation of amines by polymers containing Si-H groups. Lin et al. [27], Guerra-Contreras et al. [38], and Lei et al. [39] performed hydrosilylation of a tertiary amine, *N*,*N*-dimethylallylamine (DMAA), with PHMS-DMS in the presence of Karstedt’s catalyst, affording amine-functionalized polysiloxanes. In these studies, the synthesized polymers with amine moieties were converted to the quaternary ammonium salts (QAS) by treatment with 1-iodooctene [38] or benzyl chloride [27,39]. The prepared polymers in their salt forms showed high antibacterial activity [39]. Kanjilal et al. [40], in turn, reported the reaction of PHMS with *N*-allylcyclohexylamine in the presence of Speier’s catalyst. The modified polymers obtained were used for the fabrication of membranes on porous polyethylene supports. All of these studies employ a well-known, simple hydrosilylation process, carried out under mild conditions (90–100 °C), either with [38,39,40] or without [27] a solvent, for the preparation of amine-modified polysiloxanes.

To the best of our knowledge, studies on cross-linked polysiloxanes bearing amino or ammonium substituents are limited. Silanol-terminated siloxane copolymers modified by QA units were cross-linked or co-cross-linked with a commercial silicone elastomer using tetraethoxysilane in the presence of dibutyltin dilaurate catalyst [41]. Both types of materials exhibited high antibacterial activity [41]. Silanol-terminated siloxane copolymers containing QA groups were also cross-linked with a commercial polyisocyanate to produce antibacterial coatings [25]. Amine-functionalized PDMS with silanol chain ends, in turn, was cross-linked through both covalent and supramolecular interactions, leading to the formation of porous silicone networks in film form [42]. Based on their thermal and mechanical properties and high dielectric permittivity, these materials were suitable for pressure sensors [42]. Polysiloxane networks incorporating amino or ammonium groups are therefore attractive materials due to the wide range of their potential applications, including antibacterial coatings and films, as well as the fabrication of medical devices (e.g., drains, catheters) and implants.

In our previous work, we reported that hydrosilylation conducted in the presence of Karstedt’s catalyst allows successful incorporation of the moieties of *N*-allylaniline (Naa), *N*-allylcyclohexylamine (Nach), and *N*-allylpiperidine (Nap) to a low-molecular-weight siloxane—1,1,3,3-tetramethyldisiloxane (M_2_^H^) [43] and to linear poly(hydromethyl-siloxane) (PHMS) [44]. Our recent studies showed that it is possible to prepare Naa-functionalized PHMS networks that exhibit antibacterial properties by a hydrosilylation reaction between residual Si-H groups in the systems and Naa catalyzed by Karstedt’s complex [19].

In the present study, we decided to explore the preparation of amine-functionalized PHMS networks in more detail. To obtain such materials, we used two approaches. The first one involved two steps: the synthesis of the cross-linked PHMS (CPHMS), followed by the reaction of CPHMS with Naa, Nach, or Nap. In the second, one-step procedure, PHMS cross-linking and functionalization took place simultaneously. 1,1,3,3-tetramethyl-1,3-divinyldisiloxane (M_2_^Vi^) served as the PHMS cross-linking agent while functionalization was carried out at two molar ratios of *N*-allyl to Si-H groups.

Characterization of the obtained materials enabled us to compare the efficiency of PHMS networks functionalization by Naa, Nach, and Nap using both methods. Moreover, the influence of the type of amine and the molar ratio of reactive groups involved in the hydrosilylation reaction on the degree of network functionalization in the studied systems was determined. We hope that our research will add new and valuable knowledge to the field of hydrosilylation using Karstedt’s catalyst for the synthesis of polysiloxane networks functionalized with amino groups, the materials of versatile applications.

## 2. Results and Discussion

**Figure 1:**
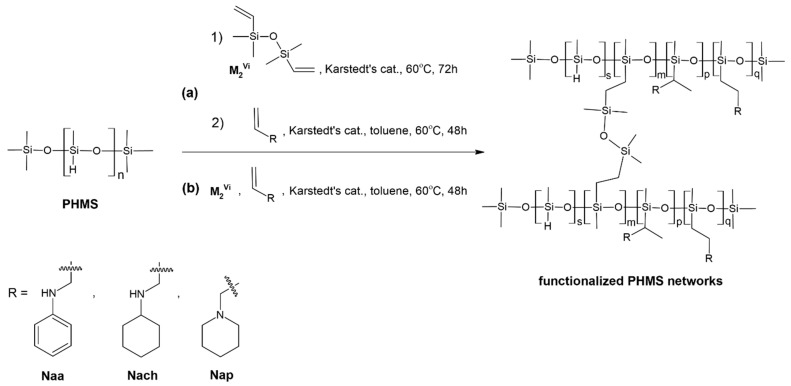
Preparation of PHMS networks functionalized with *N*-allyl amines in the work: (**a**) a two-step process, (**b**) a one-step process.

In this work, PHMS-based polysiloxane networks functionalized with *N*-allylamines (Naa, Nach, and Nap) were prepared in two ways, as presented schematically in Figure 1. The first one (route a in Figure 1) was a two-step procedure, which involved PHMS cross-linking with M_2_^Vi^ first, followed by the reaction of the obtained network with a given amine. In the second, one-step method (route b in Figure 1), all reagents were added to the reaction medium simultaneously, and PHMS cross-linking and functionalization took place at the same time. Independently of the method, the molar ratio of Si-Vinyl to Si-H groups in the polymer cross-linking process was fixed at 0.17:1 (Section 3.2), while functionalization was performed at two molar ratios of *N*-allyl to Si-H groups (the latter calculated as those remaining after or not participating in polymer cross-linking) equal to 0.5:1 and 1.5:1 (Section 3.3 and Section 3.4). Thus, in the adopted procedures, both PHMS cross-linking and network functionalization were performed by hydrosilylation, i.e., catalytic addition of Si-H to double carbon–carbon bonds [11]. It was catalyzed by a Pt complex, the so-called Karstedt’s catalyst (Section 3.2, Section 3.3 and Section 3.4).

In the following parts of the paper, the symbols: CP_Naa_1.5, CP_Nach_1.5, CP_Nap_1.5, and CP_Naa_0.5, CP_Nach_0.5, and CP_Nap_0.5 are used to denote products obtained by the two-step method, i.e., CPHMS functionalized with the appropriate *N*-allylamine at the Si-H:CH_2_=CH-CH_2_- groups molar ratios equal to 1:1.5 and 1:0.5, respectively. The symbols: FCP_Naa_1.5, FCP_Nach_1.5, FCP_Nap_1.5, FCP_Naa_0.5, FCP_Nach_0.5, and FCP_Nap_0.5 are used to denote PHMS simultaneously cross-linked using M_2_^Vi^ and functionalized with the appropriate *N*-allylamine.

As already mentioned, the main aim of the study was to compare the efficiency of two methods in the functionalization of PHMS networks by Naa, Nach, and Nap. Therefore, all the prepared materials were characterized by spectroscopic techniques (FTIR, ^29^Si MAS-NMR) and elemental analysis (determination of C, H, N contents). Additionally, to evaluate their thermal properties, thermogravimetric measurements were performed. Results of these investigations are discussed in the following sections, separately for each procedure applied.

### 2.1. Two-Step Procedure

Cross-linked PHMS (CPHMS), applied in the two-step method as the starting material for functionalization by Naa, Nach, and Nap, was prepared using a low amount of M_2_^Vi^ with respect to the polymer. This was because, for the incorporation of amines, due to steric reasons, the low cross-link density of PHMS was believed to be beneficial. Additionally, the presence of high amounts of Si-H groups, ensured by low polymer cross-link density, was required to obtain high functionalization degrees in a hydrosilylation process.

Experiments showed that equilibrium swelling in toluene (this solvent was selected because it was used in the reactions of CPHMS with amines, Section 3.3) of the prepared CPHMS was high (162% wt./wt), indicating its expected low cross-link density. Indeed, the average cross-link density calculated based on swelling measurements using Flory–Rehner’s equation [45], equal to 1.99 × 10^−3^ mol/cm^3^ (average molecular weight between cross-links of 525 g/mol), was significantly lower than that found for PHMS networks formed with higher amounts of M_2_^Vi^ in relation to the polymer (5.59 × 10^−3^ mol/cm^3^ and average molecular weight between cross-links of 187 g/mol at Si-Vi:Si-H groups molar ratio equal to 0.66:1 [14]).

**Figure 2:**
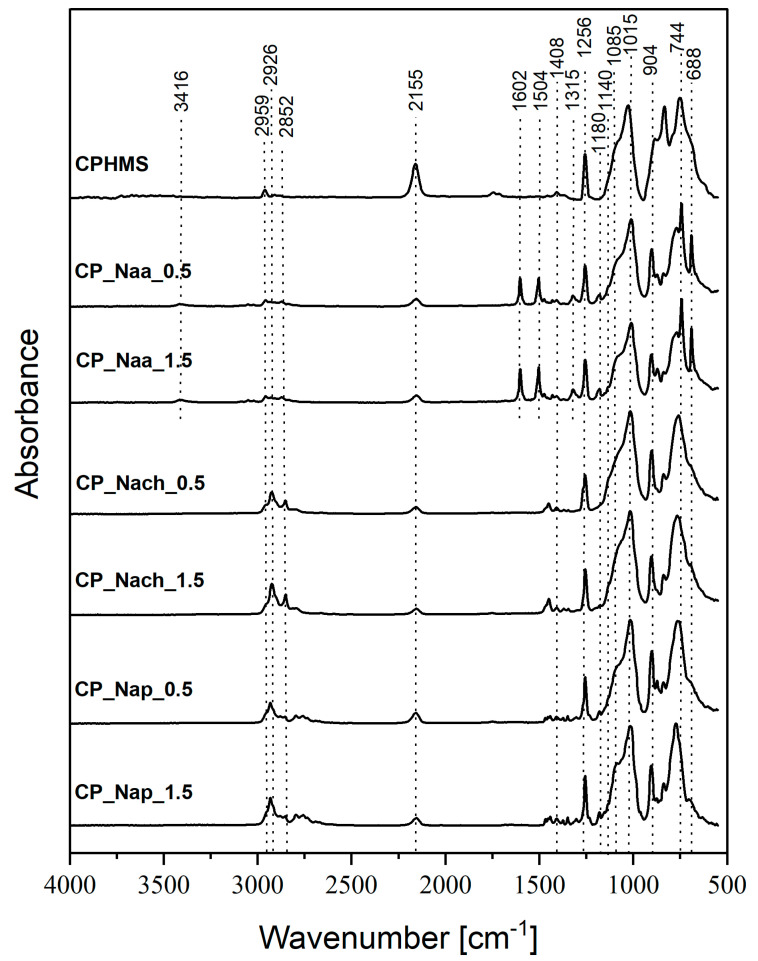
FTIR spectra of CPHMS and products of its functionalization by Naa, Nach, and Nap (two-step procedure).

**Figure 3:**
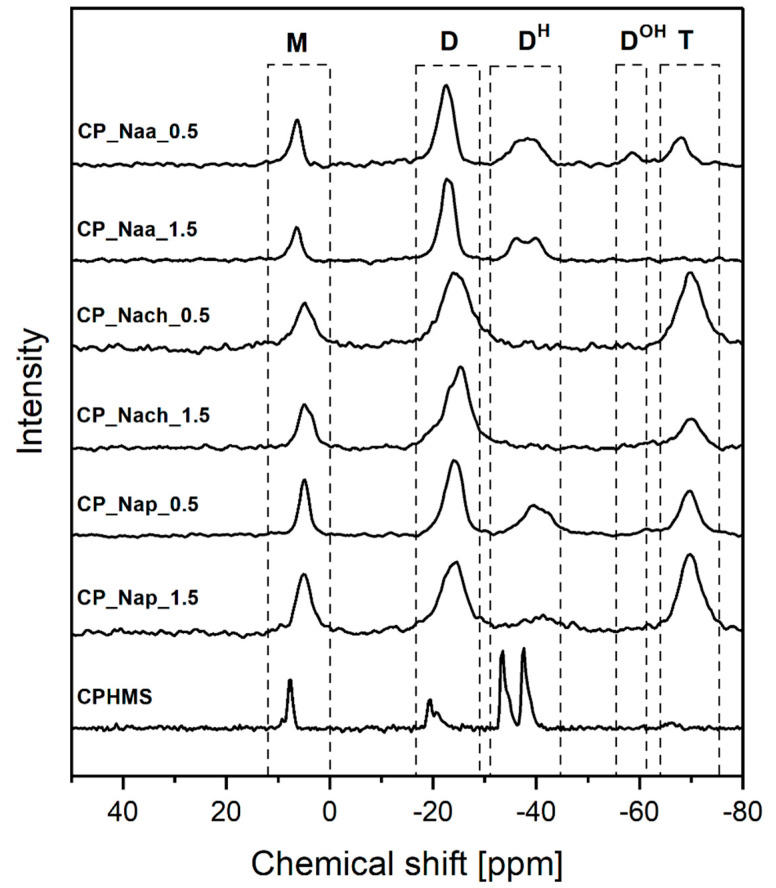
^29^Si MAS-NMR spectra of CPHMS and products of its functionalization by Naa, Nach, and Nap.

FTIR (Figure 2) and ^29^Si MAS-NMR (Figure 3) spectra confirmed that in CPMHS, Si-H groups remained. Their presence was unequivocally proved by the FTIR band at 2155 cm^−1^ due to stretching vibrations of the Si-H bond [46] and ^29^Si MAS-NMR signals in the range of chemical shift values, δ from −33.3 to −37.5 ppm corresponding to [SiO_2_CH_3_H] (D^H^) units in various chemical environments [47]. According to quantitative ^29^Si MAS-NMR spectrum analysis, D^H^ were the dominant units in CPHMS. They constituted 64% in the system (Table 1) vs. 71% calculated for PHMS cross-linked at Si-Vi:Si-H groups molar ratio used in the work (0.17:1). The discrepancy suggested that some Si-H groups of PHMS were consumed in other reactions than hydrosilylation.

Spectroscopic studies also evidenced that CPHMS was the product of hydrosilylation. Its FTIR spectrum (Figure 2) contained the bands at 2929 cm^−1^, 2852 cm^−1^, and a shoulder at 1140 cm^−1^ originating from vibrations of -CH_2_- groups in Si-CH_2_-CH_2_-Si linkages [46] formed in this process. In the ^29^Si MAS-NMR spectrum (Figure 3) these bridges were represented by two signals: at δ = 8 ppm that could be assigned to the [SiO(CH_3_)_2_(CH_2_CH_2_)] (M) units [47] and at δ = −19.5 ppm due to [SiO_2_(CH_3_)(CH_2_CH_2_)] (D) units, both generated upon hydrosilylation of M_2_^Vi^ by PHMS (Figure 1). In agreement with the stoichiometry of the cross-linking reaction, their fractions in the deconvoluted ^29^Si MAS-NMR spectrum of CPHMS were the same, equal to 16% (Table 1). This is close to 14.5% which is the value calculated for the molar ratio of Si-Vi:Si-H groups = 0.17 used in the experiments.

Occurrence of side reactions during PHMS cross-linking by M_2_^Vi^ was corroborated by the signal at δ = −65.7 ppm originating from [SiO_3_(CH_3_)] (T) units [47] revealed after deconvolution of the ^29^Si MAS-NMR spectrum of CPHMS. This signal indicated the hydrolysis of some Si-H groups of the polymer, followed by the condensation of the formed Si-OH groups. As could be judged by the low fraction of T units in the spectrum (Table 1), hydrolysis of Si-H groups proceeded to a small extent.

From both FTIR and ^29^Si MAS-NMR spectra, it could be concluded that reactions with Naa, Nach, and Nap resulted in a significant decrease in the amount of Si-H groups in the CPHMS network and concomitant increase in the amount of -CH_2_- moieties. In the FTIR spectra of the materials reacted with amines as comapared to the spectrum of CPHMS, the band at 2155 cm^−1^, attributed to Si-H bonds, showed distinctly lower intensity, while the bands at 2926 cm^−1^ and 2852 cm^−1^, originating from -CH_2_- groups, showed higher intensities (Figure 2). Similarly, ^29^Si MAS-NMR signals corresponding to D^H^ units became less intense and those corresponding to D units became more intense in the spectra of the products of the reactions as compared to those of CPHMS (Figure 3). Hence, spectroscopic investigations revealed the formation of Si-CH_2_-CH_2_-CH_2_- groups at the expense of Si-H ones, thus proving the functionalization of CPHMS by Naa, Nach, and Nap.

The presence of amines in the functionalized CPHMS was confirmed by a weak FTIR band at 1180 cm^−1^, assigned to stretching vibrations of the C-N bond [46] (Figure 2 and Figure S1). It was particularly well resolved in the spectra of CP_Nap_0.5 and CP_Nap_1.5, as well as CP_Naa_0.5 and CP_Naa_1.5 materials (Figure S1). Other bands corresponding to Naa were also observed in the spectra at 3416 cm^−1^, 1504 cm^−1^, 1602 cm^−1^, and 1315 cm^−1^ (Figure 2 and Figure S1) due to N-H stretching, aromatic C=C stretching, N-H deformational, and C-N stretching vibrations [46], respectively.

As found by quantitative FTIR spectra analysis, conversion degrees of Si-H groups ranged from 53.5% to 78.3%; they were the highest in the reactions with Nach, lower in the reactions with Naa, and the lowest in the reactions with Nap, and depended on the molar ratio of *N*-allyl to Si-H groups in the process (Table 1). According to the ^29^Si MAS-NMR technique, the sequence of D^H^ units transformation degrees—manifested by the decrease in the shares of these units in the spectra relative to that of CPHMS—was the same as that established by FTIR spectroscopy: the highest in the case of Nach, the lowest for Naa (Table 1). However, there were discrepancies between the spectra, e.g., signals of D^H^ units were not observed at all in the ^29^Si MAS-NMR spectra when CPHMS reacted with Nach (Figure 3, Table 1), even though the bands due to Si-H bonds were observed in the FTIR spectra of these materials (Figure 2, Table 1).

Nevertheless, the great advantage of ^29^Si MAS-NMR spectroscopy was that it allowed for the determination of functionalization degrees of CPHMS by Naa, Nach, and Nap. Moreover, it made it possible to discover that side reactions of Si-H groups, which proceeded independently of functionalization had an important role in most systems.

It should be mentioned here that, in contrast to PHMS cross-linking by M_2_^Vi^, functionalization of CPHMS by Naa, Nach, and Nap via hydrosilylation generated only Si-CH_2_-CH_2_-CH_2_ linkages, i.e., [SiO_2_(CH_3_)(CH_2_CH_2_CH_2_)] (D) units, while M units stayed intact in this process (Figure 1). Quantitative analysis confirmed that fractions of M units in the deconvoluted ^29^Si MAS-NMR spectra of CPHMS before and after functionalization were similar (Table 1). Therefore, the difference between the share of D units in the spectrum of a given functionalized material and that in the spectrum of CPHMS could be considered as the CPHMS functionalization degree by a given amine. Functionalization degrees thus defined were the highest in the case of Nach, lower for Naa, and the lowest for Nap (Table 1). Notably, the functionalization degree of the CP_Nap_0.5 and CP_Nap_1.5 samples differed to a low extent, while in the case of Naa and Nach, as expected, the use of a higher amount of amine resulted in distinctly higher functionalization degree (Table 1).

Side reactions of Si-H groups were marked by the signal corresponding to T units observed in most of the ^29^Si MAS-NMR spectra of the functionalized materials; it was absent only in the spectrum of the CP_Naa_1.5 sample (Figure 3). This showed that during reactions with the studied amines, a part of the Si-H groups of CPHMS were hydrolyzed to Si-OH, which then condensed to form new Si-O bonds in silsesquioxane (T) units. Amines are known as basic catalysts of such processes, serving their function even in the presence of small amounts of water [48,49].

Shares of T units were particularly high in the spectra of CPHMS functionalized with Nach and Nap (Table 1). This could be explained by the significantly higher basicity of Nach (pKa = 9.19 [50]) and Nap (pKa = 9.68 [51]) compared with that of Naa (pKa = 4.14 [52]). It is also worth noting that for all amines, lower functionalization degrees were accompanied by higher hydrolysis/condensation extents, as shown by higher fractions of T units in the ^29^Si MAS-NMR spectra (Table 1). In the spectrum of the CP_Naa_0.5 sample, which had the lowest fraction of T units, a signal at δ = −58.4 ppm, assigned to [SiO_2_(CH_3_)OH] (D^OH^) units [47] could be distinguished (Figure 3, Table 1). This indicated a low rate of condensation of silanol groups when Naa acted as a catalyst of this process, which was consistent with the low basicity of this amine.

Elemental analysis showed that the contents of nitrogen in CPHMS functionalized with Naa and Nach markedly grew as the molar ratio of the *N*-allyl to Si-H groups in the reaction increased (Table 2). The opposite was observed for Nap, but the difference in nitrogen contents between CP_Nap_0.5 and CP_Nap_1.5 was small (Table 2). These results agreed with the relationships between functionalization degrees established from the ^29^Si MAS-NMR spectra (Table 1).

To determine the influence of amino groups on thermal properties of the systems, CPHMS and the products of its functionalization by Naa, Nach, and Nap were subjected to a thermogravimetric (TG) analysis (Section 3.5). According to its results (Figure S3), CPHMS started to decompose at a lower temperature than most of the functionalized materials. Moreover, the highest rates of decomposition of CPHMS were manifested by two distinct maxima on its derivative TG (DTG) curve: of higher intensity at 198 °C and of lower intensity at 509 °C (Figure S3, Table S1). The fastest degradation of the functionalized materials, in turn, took place at 418–454 °C (Figure S3, Table S1). Hence, functionalization with amines retarded thermal decomposition of CPHMS. This effect could be caused by a decrease in the number of Si-H groups in the systems upon functionalization. Polysiloxane networks containing Si-H bonds, whose energy is low, start to disintegrate at low temperatures [53,54,55].

Residual mass at 1000 °C, determined from TG curves, was higher than that of CPHMS for the CP_Naa_0.5, CP_Nap_0.5, and CP_Nap_1.5, while for the CP_Naa_1.5, CP_Nach_0.5, and CP_Nach_1.5 samples it was lower than that of CPHMS (Table S1). This showed that thermal degradation of the former materials was less, while that of the latter—more intense than that of CPHMS. Notably, the lowest residual mass was found for the CP_Naa_1.5 and CP_Nach_1.5 samples (Table S1), which had the highest functionalization degrees (Table 1). This suggested that there could exist a certain threshold functionalization degree after which the thermal properties of the functionalized system deteriorated. To support this conclusion and to fully understand the thermal properties of the systems, more studies would be needed. They were, however, beyond the scope of the present work.

### 2.2. One-Step Procedure

In the one-step procedure, two hydrosilylation processes, namely PHMS cross-linking by M_2_^Vi^ and functionalization by the amine, proceeded simultaneously (Figure 1). It was of interest then to check how such competition influenced polymer cross-linking degree and efficiency of PHMS network modification by Naa, Nach, and Nap.

**Figure 4:**
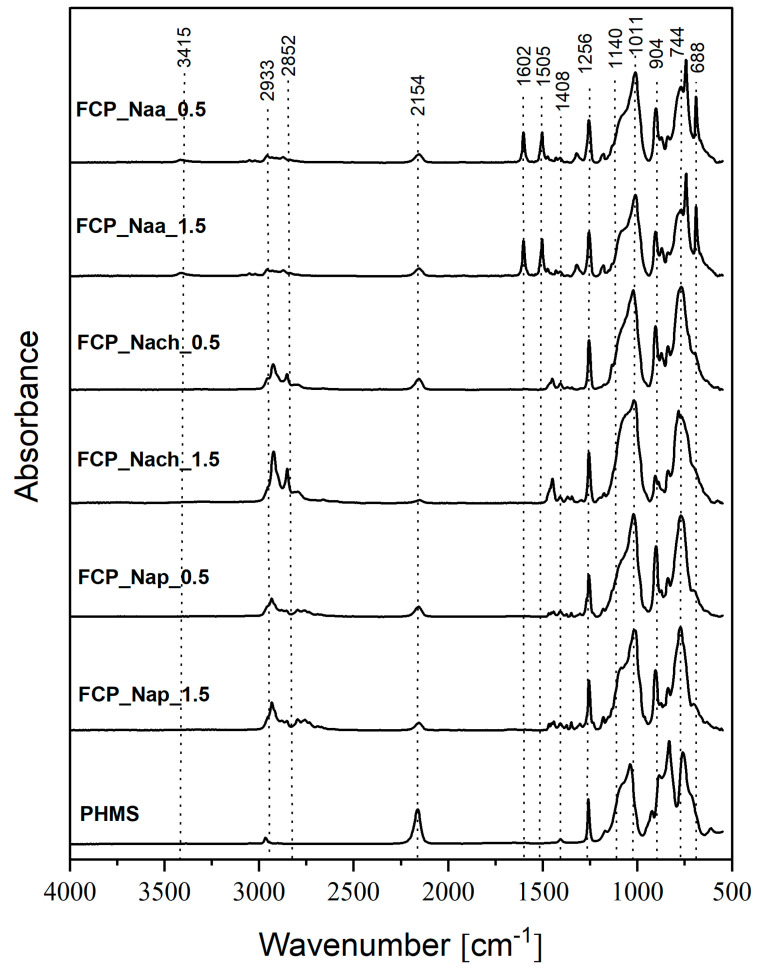
FTIR spectra of PHMS and the studied functionalized materials obtained in the one-step procedure.

All the obtained materials were solid, which was the first sign of successful cross-linking of PHMS in the conditions of the one-step process. Their FTIR spectra (Figure 4 and Figure S2) were similar to those of functionalized CPHMS (Figure 2 and Figure S1). Thus, the spectra showed the bands proving the presence of -CH_2_- bonds in Si-CH_2_-CH_2_- and Si-CH_2_-CH_2_-CH_2_- linkages (2933 cm^−1^, 2852 cm^−1^, 1140 cm^−1^) as well as the bands of groups occurring in amines (1180 cm^−1^ and in the spectra of FCP_Naa_0.5 and FCP_Naa_1.5 samples additionally 3415 cm^−1^, 1602 cm^−1^, 1505 cm^−1^, and 1315 cm^−1^). It could therefore be concluded that both PHMS cross-linking and functionalization by amines indeed took place in the systems.

In the FTIR spectra of the samples functionalized by the one-step procedure, the band at 2154 cm^−1^ originating from Si-H bonds was of lower intensity than in the spectrum of the starting PHMS (Figure 4). Conversion degrees of Si-H groups of PHMS calculated based on FTIR spectra were generally higher in the one-step (Table 3) than in the two-step (Table 1) approach. This is understood since in the one-step procedure, Si-H moieties were consumed in two processes. Similarly to the two-step protocol, among the samples simultaneously cross-linked and functionalized, the highest Si-H groups conversion degree was observed for the material obtained with Nach at the *N*-allyl to Si-H groups molar ratio equal to 1.5:1 (FCP_Naa_1.5 sample, Table 3). It could be supposed, then, that the functionalization level of this sample was the highest.

**Figure 5:**
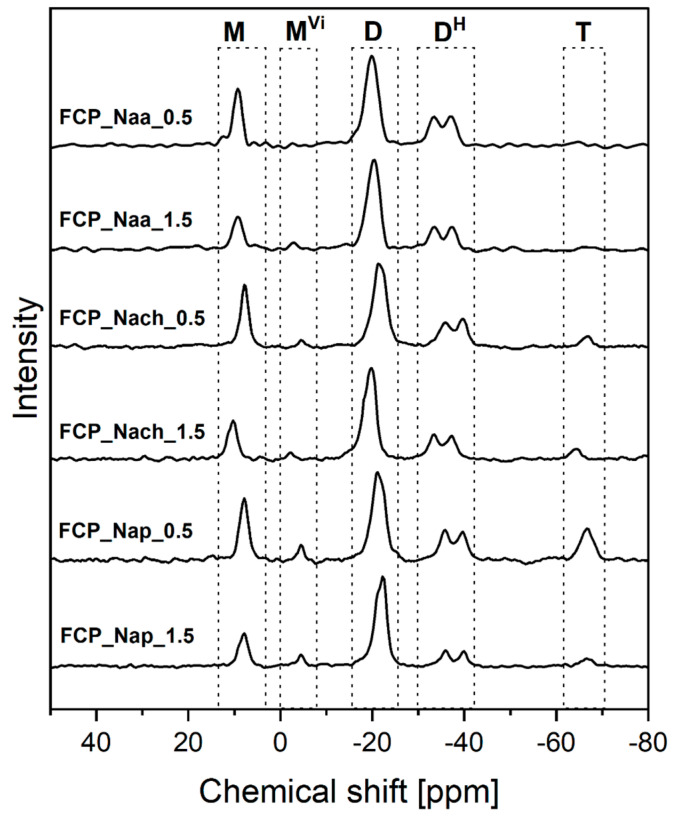
^29^Si MAS-NMR spectra of the functionalized materials obtained in the one-step procedure.

Occurrence of both PHMS cross-linking by M_2_^Vi^ and functionalization by the amines in the one-step process was also unambiguously established by ^29^Si MAS-NMR spectroscopy. Cross-linking was confirmed by the signal corresponding to M units observed in the spectra of all the samples prepared by this method (Figure 5). Its fractions (Table 3) were close to those found in the spectra of amine-modified CPHMS (Table 1), which demonstrated that the presence of amine in the one-step reaction media did not interfere with the PHMS cross-linking process. The signal of D units, showing high intensity in the spectra (Figure 5), was in turn related to the Si-CH_2_-CH_2_- and Si-CH_2_-CH_2_-CH_2_- linkages resulting from PHMS cross-linking by M_2_^Vi^ and the reactions with amines, respectively (Figure 1). Functionalization degrees, calculated as the difference between the shares of D units and M units in the spectrum (since cross-linking led to the same amounts of D and M units, Figure 1) were comparable to or higher than those determined by the analysis of the ^29^Si MAS-NMR spectra of the networks modified by Naa, Nach, Nap using the two-step approach (Table 3 vs. Table 1). In all cases, the functionalization degree increased with increasing molar ratio of *N*-allyl to Si-H groups (Table 3). The highest functionalization degree was shown by the FCP_Nach_1.5 sample, as indicated also by FTIR spectra. Particularly high functionalization degrees of FCP_Nap_0.5 and FCP_Nap_1.5 samples, similar to those of the CP_Nap_0.5 and CP_Nap_1.5 materials, should be noted. Clearly, for the functionalization by Nap, the one-step process was advantageous.

^29^Si MAS-NMR spectra of all samples prepared by the one-step method contained the signals of D^H^ units (Figure 5). In most of them, a signal of low intensity and low share corresponding to [SiO(CH_3_)_2_(CH=CH_2_)] (M^Vi^) [47] units was present (Figure 5, Table 3). The FCP_Naa_0.5 material was the exception as its spectrum did not contain this signal (Figure 5, Table 3). M^Vi^ units could result either from an incomplete removal of the unreacted M_2_^Vi^ after the reactions or from the occurrence of pending vinyl groups in the systems. Pending reactive groups are known to remain in cross-linked polysiloxanes [56].

Importantly, ^29^Si MAS-NMR spectroscopy showed that hydrolysis of Si-H and condensation of the generated Si-OH groups in the one-step functionalization processes were not as significant as in the two-step one. Signals corresponding to T units were weak and their shares were low (Figure 5, Table 3). In the spectra of the materials obtained with Naa, the weakest base of the applied amines (Section 2.1), no signal of T units could be distinguished (Figure 5). On the other hand, the highest share of T units was in the spectrum of the FCP_Nap_0.5 sample of relatively low functionalization degree (Table 3). Thus, it could be concluded that in the one-step functionalization processes, side reactions of Si-H groups were strongly impeded.

Elemental analysis confirmed incorporation of amines into the systems. All samples contained nitrogen, and its amounts were high (Table 4).

TG measurements were conducted only for the samples obtained by the one-step procedure at the ratio of *N*-allyl:Si-H groups equal to 1.5:1 (Section 3.5). The results showed that, similarly to those prepared using the two-step procedure, the materials functionalized simultaneously with PHMS cross-linking were thermally stable up to 350–400 °C (Figure S4). According to DTG curves, their fastest decomposition occurred at 416–457 °C (Figure S4, Table S1). Residual masses at 1000 °C were comparable to, or lower than, those of CPHMS; differences were also observed in comparison with the respective samples prepared by the two-step method (Table S1). However, thermal phenomena occurring in the systems were complex, and the differences could not be explained by different functionalization degrees of the materials. Thus, again, more studies are needed to understand the reasons for the observed results.

### 2.3. Two-Step and One-Step Procedures—Comparison Based on Conversion of Si-H Groups

As demonstrated in Section 2.1 and Section 2.2, ^29^Si MAS-NMR spectroscopy proved very useful in establishing the types and relative intensities of the processes occurring in the studied systems. However, cross-linking of PHMS by M_2_^Vi^ generates two types of silicon units: M and D (Figure 1). Because of this, the shares of the signals in the ^29^Si MAS-NMR spectra collected in Table 1 and Table 3 do not show directly the fractions of the polymer’s Si-H groups involved in individual reactions.

A straightforward comparison of the materials prepared by the two-step and one-step procedures based on the conversion degrees of Si-H groups of PHMS in the reactions occurring in the systems is more convenient. Therefore, using the ^29^Si MAS-NMR spectroscopic data presented in Table 1 and Table 3, we performed calculations as follows:

1. Taking into account that M units originated exclusively from the cross-linking process (Figure 1), the fraction of Si-H groups participating in this reaction was assumed to be equal to the share of the signal attributed to M units in the ^29^Si MAS-NMR spectrum of a given sample.
2. The share of the signal corresponding to Si-H groups functionalized with an amine was assumed to be equal to the difference between the shares of D and M units in the ^29^Si MAS-NMR spectrum of a given sample. This was because the same number of M and D units were formed upon polymer cross-linking (Figure 1). Thus, excessive D units were attributed to the functionalization process.
3. The sum of the fractions of the Si-H groups not taking part in PHMS cross-linking was calculated as the difference: [100% − the share of M units in the ^29^Si MAS-NMR spectrum of a given sample]. To obtain fractions of Si-H groups involved in individual reactions or staying intact, this difference was divided into parts proportional to the shares of the respective signals in the ^29^Si MAS-NMR spectrum of a given sample.
4. Additionally, conversion degrees of the Si-H groups preserved in the materials after polymer cross-linking in individual reactions were calculated. This was performed as in point 3 but assuming the difference: [100% − the share of M units in the ^29^Si MAS-NMR spectrum of a given sample] as 100%.

Results of the calculations (Table 5) confirmed that the one-step method was more advantageous than the two-step method. Fractions of Si-H groups involved in the functionalization process in the simultaneous procedure, ranging from 31 to 60%, in most cases were markedly higher than those found for CPHMS reacting with amines, ranging from 21 to 59%. Simultaneously, fractions of Si-H groups converted to T units via side reactions were significantly lower in the one-step as compared to the two-step method (the CP_Naa_1.5 sample with no T units was an exception). It should be noted here that in the CP_Nach_0.5, CP_Nap_0.5, and CP_Nap_1.5 systems, side processes prevailed over functionalization since more Si-H moieties transformed to T units (32–49%) than were functionalized (21–33%).

## 3. Materials and Methods

### 3.1. Materials

PHMS (viscosity: 35–45 cSt, average molecular weight determined by ^29^Si NMR spectrum of PHMS solution in CDCl_3_: 3800 g/mol) was purchased from ABCR (Karlsruhe, Germany) and vacuum-dried (pressure: ~10^−2^ mbar) at 60 °C for 2 h before use. 1,1,3,3-tetramethyl-1,3-divinyldisiloxane (M_2_^Vi^) was purchased from ABCR (Karlsruhe, Germany) and used in the work without purification. Platinum(0)-1,1,3,3-tetramethyl-1,3-divinyldisiloxane complex (Karstedt’s catalyst) was supplied by Sigma-Aldrich (Poznań, Poland) as a solution in xylene (2 wt.% of Pt) and used in the work without purification. Naa and Nach were purchased from Sigma-Aldrich (Poznań, Poland) and purified by vacuum distillation before use. Nap was synthesized using the procedure presented in Ref. [43]. Toluene was supplied by Avantor (Gliwice, Poland) and was distilled from sodium-benzophenone before use. Piperidine, sodium hydride 60% mixture in mineral oil, and allyl bromide applied in the preparation of Nap, were purchased from ABCR (Karlsruhe, Germany), Sigma-Aldrich (Poznań, Poland), and Avantor (Gliwice, Poland), respectively, and used in the work without purification. Diethyl ether was purchased from Avantor (Gliwice, Poland) and, before use, dried using benzophenone and sodium, and then distilled under Ar.

### 3.2. Cross-Linking of PHMS with M_2_^Vi^ (CPHMS)

PHMS was cross-linked with M_2_^Vi^ by hydrosilylation performed in the presence of Karstedt’s catalyst. Molar ratio of Si-H groups from the polymer to CH_2_=CH- groups from the cross-linking agent equal to 1:0.17 was applied.

In total, 20 g (0.0053 mol; 0.32 mol of Si-H groups) of PHMS (previously dried on a vacuum line) was introduced into a round-bottom flask, to which 6.14 mL (0.027 mol) M_2_^Vi^ and Karstedt’s catalyst solution (0.53 × 10^−6^ mol of Pt) were added. The volume of the catalyst solution was calculated assuming that the molar ratio of Si-H groups (derived from hydrogen siloxane) to Pt (present in the catalyst) was equal to 1 × 10^−5^. The reaction was carried out for 72 h under argon at 60 °C. After the reaction, the product was dried on a vacuum line. The cross-linked PHMS was ground and tested for swelling in toluene.

### 3.3. Functionalization of CPHMS with Nitrogen-Containing N-Allyl Compounds

CPHMS was functionalized with *N*-allyl compounds by hydrosilylation performed in the presence of Karstedt’s catalyst. Molar ratios of Si–H groups remaining in the material after the cross-linking process to CH_2_=CH-CH_2_- groups from the nitrogen-containing compound equal to 1:1.5 and 1:0.5 were applied. The reactions were carried out under the optimal conditions established for the functionalization of PHMS with *N*-allylamines, as described in Ref. [44].

In a typical run, 2.0 g (0.0267 mol of Si-H groups) of CPHMS, the measured amount of selected *N*-allyl compound (Naa, Nach, or Nap), 5 mL of toluene, and Karstedt’s catalyst solution (0.17 × 10^−6^ mol of Pt) were successively placed in the flowing Ar atmosphere in a Schlenk flask. Then the flask was closed, put in an oil bath, and its content, under magnetic stirring, was heated to 60 °C. The reaction was carried out at this temperature for 48 h. After this time, the materials were washed a few times with anhydrous toluene on a Büchner funnel in order to remove all unreacted amine from the sample. Then the obtained products were dried on a vacuum line.

In the reactions carried out at Si-H:CH_2_=CH-CH_2_- groups molar ratio equal to 1:1.5, 5.43 mL of Naa, 5.78 mL of Nach, and 5.87 mL of Nap (0.04 mol) were applied. For the ratio 1:0.5, the amounts of *N*-allyl compounds were as follows: 1.82 mL of Naa, 1.94 mL of Nach, and 1.97 mL of Nap (0.0134 mol).

### 3.4. Simultaneous Functionalization of PHMS with Nitrogen-Containing N-Allyl Compounds and Cross-Linking Using M_2_^Vi^

PHMS functionalization with simultaneous cross-linking using M_2_^Vi^ was performed for the molar ratio of Si-H groups in polymer to CH_2_=CH- groups in M_2_^Vi^ equal to 1:0.17 and two different molar ratios of the remaining Si-H group in PHMS to CH_2_=CH-CH_2_- groups in the organic compound equal to 1:1.5 and 1:0.5. The reactions were carried out under the optimal conditions established for the functionalization of PHMS with *N*-allylamines, as described in Ref. [44].

In a typical run, 2.0 g (0.00053 mol; 0.032 mol of Si-H groups) of PHMS (previously dried on a vacuum line) was introduced into a round-bottom flask. Then, 5 mL of toluene, 0.614 mL (0.0027 mol) of M_2_^Vi^, the measured amount of *N*-allyl amine, and Karstedt’s catalyst solution (0.17 × 10^−6^ mol of Pt) were added subsequently. The amounts of Naa, Nach, and Nap used in these reactions were equal to the amounts used to functionalize 2 g of CPHMS. The reaction was carried out in an argon atmosphere for 48 h, at 60 °C. The resulting products were washed a few times with anhydrous toluene on a Büchner funnel in order to remove all unreacted amine from the sample. Then the obtained products were dried on a vacuum line.

### 3.5. Characterization Methods

Equilibrium swelling of the prepared materials was determined in toluene. To the weighed amount of the studied sample, an excess of the solvent was added. After 36 h, the excess solvent was separated, and the swollen sample was weighed. Swelling degrees reported in this work were calculated as (m_s_ − m_0_)/m_0_ ratios, where m_s_ is the weight of the swollen sample and m_0_ is the weight of the sample subjected to swelling.

Fourier transform infrared (FTIR) spectra were recorded on a FTIR (BIO-RAD Excalibur, Bio-Rad Laboratories, Inc., Hercules, CA, USA) spectrometer, equipped with a horizontal zinc selenide (ZnSe) ATR sampling accessory. Spectra were obtained after collecting 32 scans in the 4000 to 550 cm^−1^ range; the incident beam angle was equal to 45°. Spectra analyses were conducted in the Opus 7.2. program after correcting their baselines. Quantitative analysis involved calculating the ratios of integral intensities of the band due to Si-H bond stretching vibrations at ~2155 cm^−1^ and symmetric bending vibrations of the Si-CH_3_ group at ~1256 cm^−1^ in the FTIR spectra of the obtained materials. Since Si-CH_3_ groups do not participate in the hydrosilylation reaction, the intensity of this FTIR band remained unchanged in the spectra. The Si-H/Si-CH_3_ band area ratio found for the starting compounds (PHMS or CPHMS) was assumed to constitute 100% and the corresponding ratios for the final reaction products were expressed as a percentage of this initial value, given in the work as conversion degrees of Si-H groups.

Elemental analyses were taken on a Vario El III analyzer (Elementar Analysensysteme GmbH, Langenselbol, Germany) after combustion of the analyzed sample in oxygen at 1150 °C. Contents of C, H, and N in the samples reported in the work are the average values of two analyses. The contents of Si and O in the samples were calculated as the difference: 100% − Σ %C, %H, %N.

High resolution, solid state ^29^Si MAS-NMR spectra were measured on a Tecmag APOLLO pulse NMR spectrometer (Tecmag, Inc., Houston, TX, USA) at the magnetic field of 7.05 T generated by the Magnex wide bore superconducting magnet. A Bruker HP-WB 73A high-speed MAS probe (Billerica, MA, USA) equipped with the 4 mm zirconia rotor and KEL-F cap was used to record the MAS spectra at the spinning speed of 4 kHz. The spectra were measured at 59.515 MHz, using a single 3 μs radio-frequency (rf) pulse, corresponding to a π/2 flipping angle. The acquisition delay used in accumulation was 30 s, and 128–384 scans were acquired, depending on the signal strength. The spectra were normalized to the same mass and number of accumulations. The frequency scale in ppm was referenced to the ^29^Si resonance of tetramethylsilane (TMS).

Thermogravimetric (TG) studies were performed using a TG analyzer TGA 550 Discovery (TA Instruments, New Castle, DE, USA). In a typical procedure, 10–11 mg of the sample was placed in a standard Pt crucible and heated in a N_2_ atmosphere at the rate of 10 °C/min. The investigations were conducted in the temperature range of 40–1000 °C for all samples prepared by the two-step method and only for those obtained with higher amounts of amines by the one-step method.

## 4. Conclusions

Studies conducted in this work show that PHMS networks functionalized with Naa, Nach, and Nap can be successfully prepared by hydrosilylation reactions using Karstedt’s catalyst. Although both the two-step and one-step methods developed in this work can be employed for their synthesis, the latter should be preferred. This is because it yields higher network functionalization degrees, and significantly lower contributions from side processes occur when PHMS is cross-linked and functionalized simultaneously.

It was found that side processes (hydrolysis of Si-H groups and condensation of Si-OH groups formed) are more pronounced when amines of higher basicity are applied, indicating that amines are catalysts of these transformations. Moreover, they are significant in the systems of low functionalization degrees, which suggests that they occur when functionalization is difficult. This explains why the extent of side reactions was high in the two-step procedure, which involved functionalization of the cross-linked PHMS that—because of steric reasons—was challenging.

We hope that our work will be of interest to the researchers who seek materials suitable for versatile applications. We also believe that our results can serve as guidelines for the preparation of other organofunctional cross-linked polysiloxanes.

## Figures and Tables

**Table 1:** Conversion degrees of Si-H groups evaluated by FTIR and results of ^29^Si MAS-NMR spectra analysis.

Sample	Conversion Degree of the Si-H Groups [%] ^a^	^29^Si MAS-NMR: Chemical Shift in the Signal ^b^ [ppm] (Share [%], Δ-Share Difference with Respect to the Starting Spectrum)					
		M Units	M^Vi^ Units	D Units	D^H^ Units	D^OH^ Units	T Units
CPMHS	-	8.0 (16)	-	−19.6 (16)	−33.3; −37.5 (64)	-	−65.7 (4)
CP_Naa_0.5	64.0	8.9 (15, Δ = −1)	-	−20.1 (40, Δ = +24)	−35.7 (25, Δ = −39)	−58.4 (5)	−67.4 (15, Δ = +11)
CP_Naa_1.5	69.1	8.9 (16, Δ = 0)	-	−20.5 (56, Δ = +40)	−33.7; −37.4 (28, Δ = −36)	-	-
CP_Nach_0.5	74.8	8.2 (18, Δ = +2)	-	−21.1 (44, Δ = +28)	-	-	−66.5 (38, Δ = +34)
CP_Nach_1.5	78.3	8.1 (18, Δ = +2)	-	−21.4 (62, Δ = +46)	-	-	−66.6 (20, Δ = +16)
CP_Nap_0.5	62.8	8.4 (15, Δ = −1)	-	−20.6 (36, Δ = +20)	−36.6 (23, Δ = −41)	-	−66.1 (26, Δ = +22)
CP_Nap_1.5	53.5	8.4 (17, Δ = +1)	-	−20.4 (34, Δ = +18)	−37.2 (15, Δ = −49)	-	−66.4 (34, Δ = +30)

^a^ Calculation based on FTIR spectra according to the procedure described in Section 3.5. ^b^ Symbols denote: M–[SiO(CH_3_)_2_(CH_2_CH_2_)] units, M^Vi^–[SiO(CH_3_)_2_(CH=CH_2_)] units, D–[SiO_2_(CH_3_)(CH_2_CH_2_)], [SiO_2_(CH_3_)(CH(CH_3_))] units, D^H^–[SiO_2_(CH_3_)H] units, D^OH^–[SiO_2_(CH_3_)OH] units, and T–[SiO_3_(CH_3_)] units.

**Table 2:** Results of elemental analysis of the functionalized materials obtained in the two-step procedure.

Sample	Contents of Elements [wt.%]				
	N		C	H	SiO ^c^
	calc. ^a^	Found ^b^			
CP_Naa_0.5	4.06	3.58 (88.2)	44.71	8.25	43.46
CP_Naa_1.5	5.89 (6.87)	4.94 (84.3)	52.37	8.73	33.97
CP_Nach_0.5	3.99	3.28 (82.2)	42.08	12.53	42.11
CP_Nach_1.5	5.71 (6.67)	5.18 (82.7)	51.90	15.59	27.33
CP_Nap_0.5	4.16	3.03 (72.8)	39.26	12.51	45.20
CP_Nap_1.5	6.07 (7.15)	2.57 (42.3)	34.68	11.63	51.12

^a^ Without brackets—the maximum possible nitrogen content [%] resulting from the stoichiometry of the reaction; in brackets—resulting from the amount of the *N*-allyl compound used in the reaction. ^b^ In brackets: fractions of N incorporated into the polymer, calculated as (N_found_/N_calc._)·100%. ^c^ %SiO = 100% − Σ %C, %H, %N.

**Table 3:** Conversion degrees of Si-H groups determined from FTIR spectra and results of ^29^Si MAS-NMR spectra analysis of the materials functionalized by the one-step procedure.

Sample	Conversion Degree of the Si-H Groups [%] ^a^	^29^Si MAS-NMR: Chemical Shift in the Signal ^b^ [ppm] (Share [%]; Functionalization Degree ^c^)					
		M Units	M^Vi^ Units	D Units	D^H^ Units	D^OH^ Units	T Units
FCP_Naa_0.5	79.5	8.6 (21)	-	−20.5 (51; 30)	−34.1; −37.9 (28)	-	-
FCP Naa_1.5	80.3	8.6 (17)	−3.6 (2)	−20.8 (57; 40)	−34.1; −38.0 (24)	-	-
FCP_Nach_0.5	74.0	8.1 (21)	−4.3 (1)	−21.3 (49; 28)	−35.5; −39.4 (25)	-	−65.8 (4)
FCP_Nach_1.5	91.7	8.1 (16)	−4.1 (2)	−21.2 (64; 48)	−35.0; −38.8 (14)	-	−65.9 (4)
FCP_Nap_0.5	76.0	8.2 (19)	−4.1 (3)	−21.0 (43; 24)	−35.4; −39.2 (20)	-	−66.3 (15)
FCP_Nap_1.5	83.3	8.4 (16)	−4.1 (5)	−21.5 (60; 44)	−35.5; −39.5 (12)	-	−66.3 (5)

^a,b^ As described in the caption of Table 1, M^Vi^ denotes [SiO(CH_3_)_2_(CH=CH_2_)] units. ^c^ Share of D units resulting from functionalization by the amine, calculated as the difference between the total share of D units in the spectrum and the share of M units.

**Table 4:** Results of elemental analysis of the materials obtained in the one-step procedure.

Sample	Contents of Elements [wt.%]				
	N		C	H	SiO ^c^
	calc. ^a^	Found ^b^			
FCP_Naa_0.5	4.06	4.02 (99.0)	50.54	8.30	37.14
FCP_Naa_1.5	5.86 (6.87)	5.61 (95.7)	59.86	8.94	25.59
FCP_Nach_0.5	3.99	3.73 (93.5)	47.67	10.99	37.61
FCP_Nach_1.5	5.71 (6.67)	5.35 (93.7)	58.62	12.30	23.73
FCP_Nap_0.5	4.16	3.23 (77.6)	41.25	9.98	45.54
FCP_Nap_1.5	6.07 (7.15)	4.95 (81.5)	50.49	11.04	33.52

^a^ Without brackets—the maximum possible nitrogen content [%] resulting from the stoichiometry of the reaction; in brackets—resulting from the amount of the *N*-allyl compound used in the reaction. ^b^ In brackets: fractions of N incorporated into the polymer, calculated as (N_found_/N_calc._)·100%; ^c^ %SiO = 100% − Σ %C, %H, %N.

**Table 5:** Fractions of Si-H groups participating in various reactions and left unreacted during two-step and one-step functionalization processes were calculated based on ^29^Si MAS-NMR data.

Type of Si-H Groups	Fraction in the Sample [%] (Conversion Degree ^a^ [%])					
	CP_Naa_0.5 vs. FCP_Naa_0.5	CP_Naa_1.5 vs. FCP_Naa_1.5	CP_Nach_0.5 vs. FCP_Nach_0.5	CP_Nach_1.5 vs. FCP_Nach_1.5	CP_Nap_0.5 vs. FCP_Nap_0.5	CP_Nap_1.5 vs. FCP_Nap_1.5
Cross-linked	15 vs. 21	16 vs. 17	18 vs. 21	18 vs. 16	15 vs. 19	17 vs. 16
Functionalized	30.5 (36) vs. 41 (52)	49 (59) vs. 50 (61)	33 (41) vs. 38 (48)	56 (69) vs. 60 (71)	25 (30) vs. 31 (39)	21 (26) vs. 56 (67)
Converted to D^OH^ units	6 (7) vs. 0	0 vs. 0	0 vs. 0	0 vs. 0	0 vs. 0	0 vs. 0
Converted to T units	18 (21) vs. 0	0 vs. 0	49 (59) vs. 6 (7)	26 (31) vs. 5 (6)	32 (37) vs. 20 (24)	43 (51) vs. 6.5 (8)
Converted to M^Vi^ units	0 vs. 0	0 vs. 3	0 vs. 1	0 vs. 2	0 vs. 4	0 vs. 6.5
Unreacted	30.5 vs. 38	35 vs. 30	0 vs. 34	0 vs. 17	28 vs. 26	19 vs. 15

^a^ Related to the Si-H groups remaining after polymer cross-linking.

## Data Availability

The original contributions presented in this study are included in the article/Supplementary Materials. Further inquiries can be directed to the corresponding authors.

## Supplementary Materials

The following supporting information can be downloaded at: https://www.mdpi.com/article/10.3390/ijms262211133/s1.
