# Supplementary material for: Polysiloxane Networks Modified by Nitrogen-Containing Organic Compounds
Source: Int J Mol Sci. 2025 Nov 18;26(22):11133. doi: 10.3390/ijms262211133 (PMC12652986; doi:10.3390/ijms262211133)
Supplement: Supplementary file 1 [file ijms-26-11133-s001.zip › ijms-3960104-supplementary.pdf]

Polysiloxane Networks Modified by Nitrogen-Containing Organic Compounds

Aleksandra Chechelska-Noworyta[1], Maria Owińska[1*], Magdalena Hasik[1*]

[1] Faculty of Materials Science and Ceramics, AGH University of Krakow, Kraków, Poland

* Correspondence: mhasik@agh.edu.pl; owinska@agh.edu.pl

Supplementary Materials

Contents:

Figure S1. FTIR spectra of CPMHS and products of its functionalization by Naa, Nach and Nap (two-step procedure) showing the range of 1750–550 cm^{-1}.

Figure S2. FTIR spectra of the studied materials obtained in the one-step procedure showing the range of 1750–550 cm^{-1}.

Figure S3. TG and DTG curves of CPHMS and products of its functionalization by Naa, Nach and Nap (two-step procedure).

Figure S4. TG and DTG curves of selected functionalized materials obtained in the one-step procedure.

Table S1. Residual mass at 1000°C and temperatures of maximum mass losses determined by TG and DTG investigations of CPHMS and functionalized networks studied in the work.

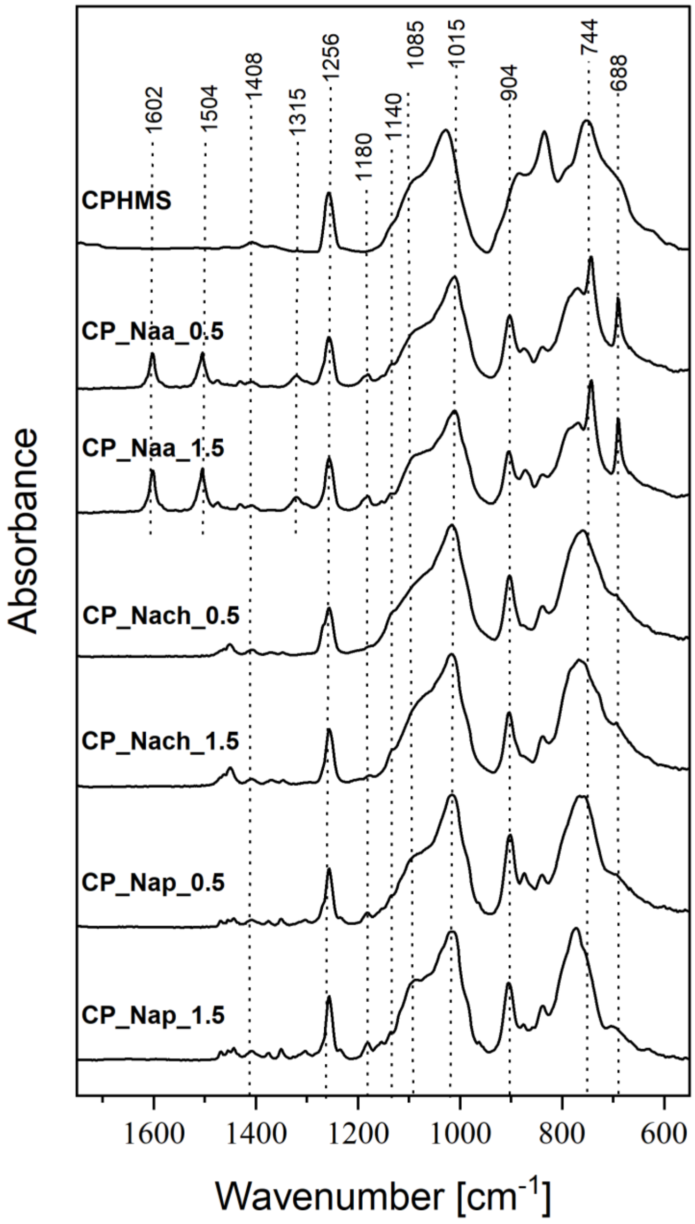

Figure S1. FTIR spectra of CPMHS and products of its functionalization by Naa, Nach and Nap (two-step procedure) showing the range of 1750–550 cm^{-1}.

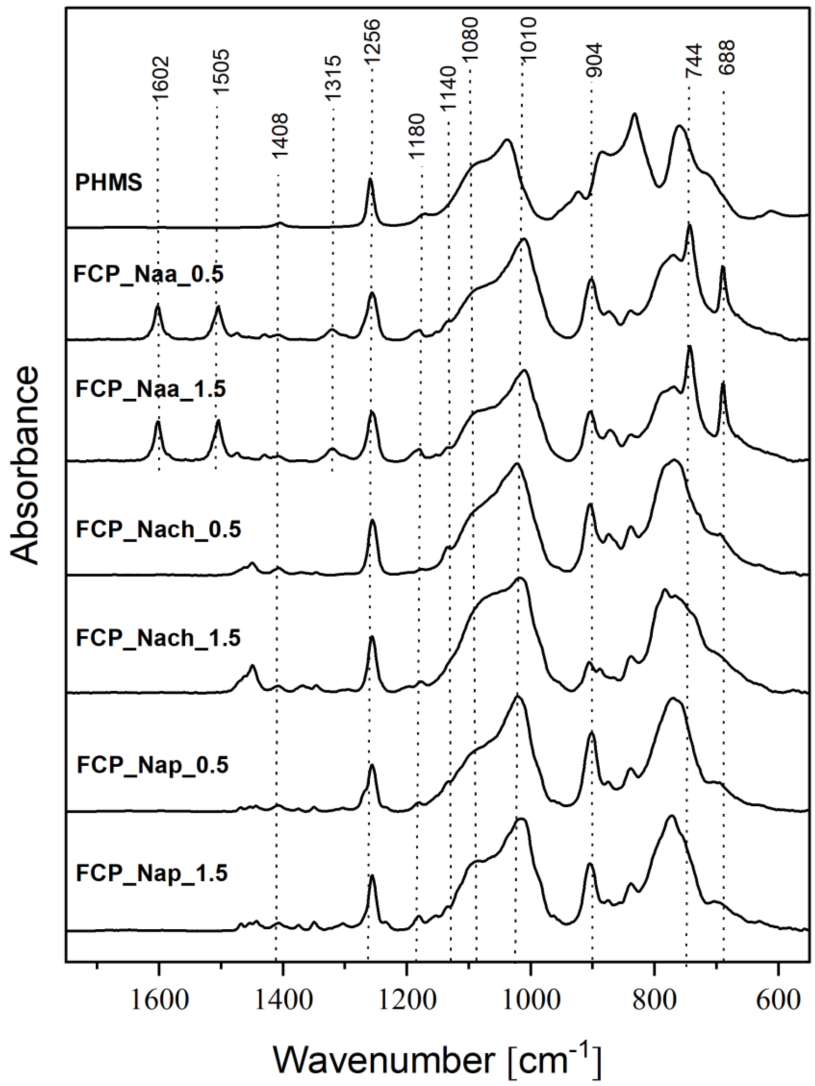

Figure S2. FTIR spectra of the studied materials obtained in the one-step procedure showing the range of 1750–550 cm^{-1}.

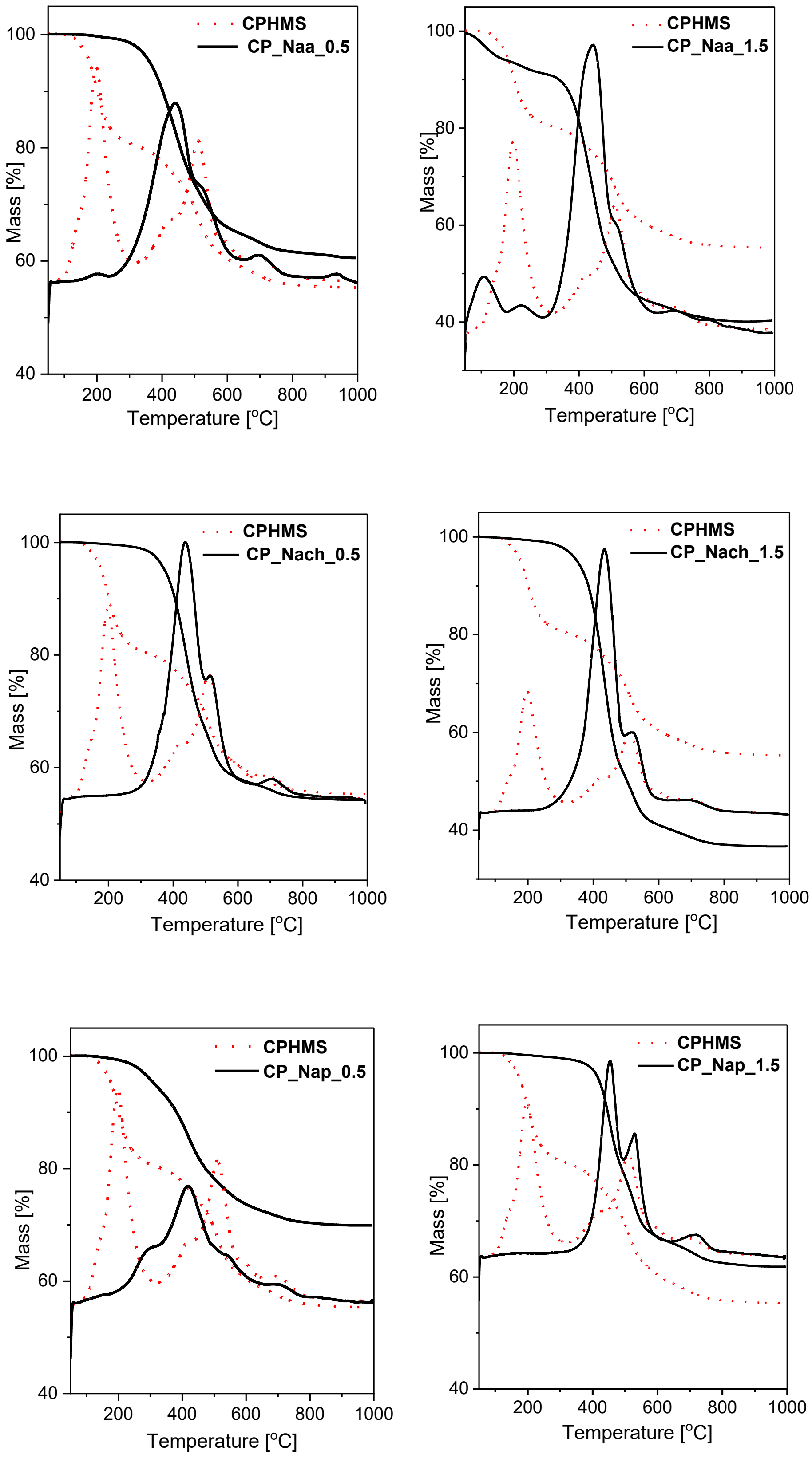

Figure S3. TG and DTG curves of CPHMS and products of its functionalization by Naa, Nach and Nap (two-step procedure).

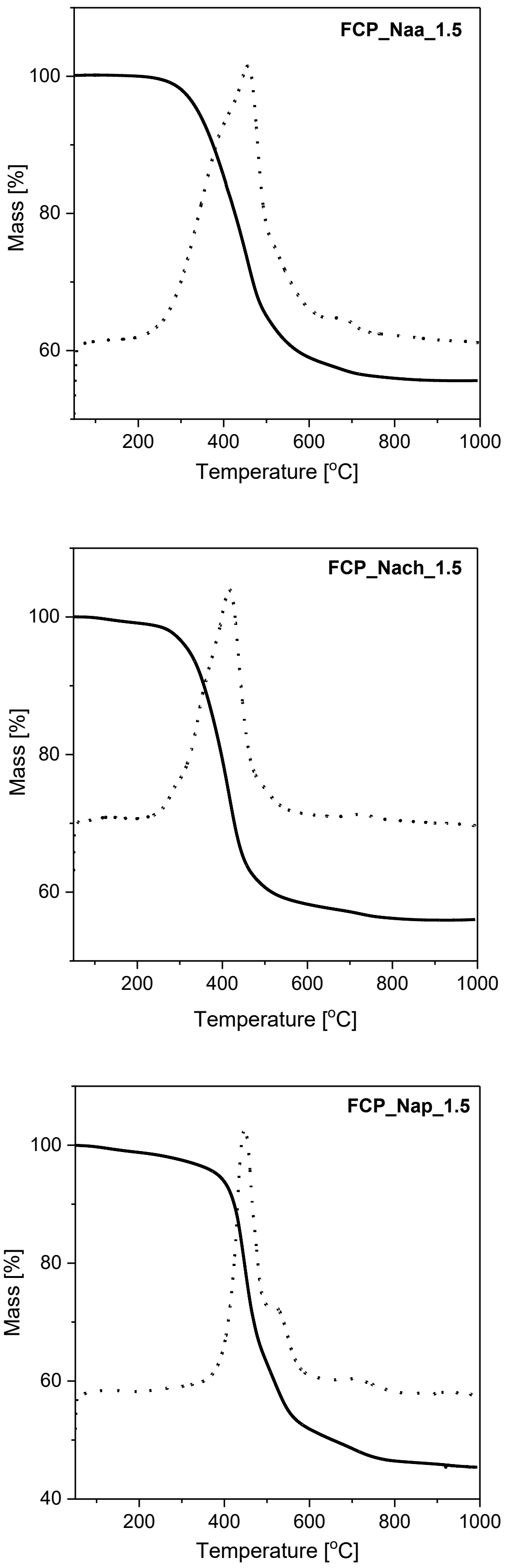

Figure S4. TG and DTG curves of selected functionalized materials obtained in the one-step procedure.

Table S1. Residual mass at 1000°C and temperatures of maximum mass losses determined by TG and DTG investigations of CPHMS and functionalized networks studied in the work.

**Sample**	**Residual mass [wt.%]**	**Temperature of maximum mass loss (T_{max}) [°C]**
Two-step procedure		
CPHMS	55.3	198, 509
CP_Naa_0.5	60.5	441
CP_Naa_1.5	40.3	444
CP_Nach_0.5	54.2	437
CP_Nach_1.5	36.7	434
CP_Nap_0.5	69.9	418
CP_Nap_1.5	61.9	454
One-step procedure		
FCP_Naa_1.5	55.6	457
FCP_Nach_1.5	56.0	416
FCP_Nap_1.5	45.4	448
